# U‐shaped association between serum IGF2BP3 and T2DM: A cross‐sectional study in Chinese population

**DOI:** 10.1111/1753-0407.13378

**Published:** 2023-03-09

**Authors:** Xiaoying Wu, Wei Wang, Shujin Fan, Lili You, Feng Li, Xiaoyun Zhang, Hongshi Wu, Juying Tang, Yiqin Qi, Wanting Feng, Li Yan, Meng Ren

**Affiliations:** ^1^ Department of Endocrinology Sun Yat‐sen Memorial Hospital, Sun Yat‐sen University Guangzhou People's Republic of China; ^2^ Department of Endocrinology National Center of Gerontology, Beijing Hospital, Peking University Fifth School of Clinical Medicine Beijing China

**Keywords:** gene expression omnibus, insulin‐like growth factor 2 mRNA‐binding protein (IGF2BP)3, N6‐methyladenosine (m^6^A) RNA methylation, type 2 diabetes mellitus, 基因表达数据库, 胰岛素样生长因子2 mRNA结合蛋白3 (IGF2BP3), N6 ‐甲基腺苷(m^6^A), RNA甲基化, 2型糖尿病

## Abstract

**Objective:**

To clarify the expression of N6‐methyladenosine (m^6^A) modulators involved in the pathogenesis of type 2 diabetes mellitus (T2DM). We further explored the association of serum insulin‐like growth factor 2 mRNA‐binding proteins 3 (IGF2BP3) levels and odds of T2DM in a high‐risk population.

**Methods:**

The gene expression data set GSE25724 was obtained from the Gene Expression Omnibus, and a cluster heatmap was generated by using the R package ComplexHeatmap. Differential expression analysis for 13 m^6^A RNA methylation regulators between nondiabetic controls and T2DM subjects was performed using an unpaired *t* test. A cross‐sectional design, including 393 subjects (131 patients with newly diagnosed T2DM, 131 age‐ and sex‐matched subjects with prediabetes, and 131 healthy controls), was carried out. The associations between serum IGF2BP3 concentrations and T2DM were modeled by restricted cubic spline and logistic regression models.

**Results:**

Two upregulated (IGF2BP2 and IGF2BP3) and 5 downregulated (methyltransferase‐like 3 [METTL3], alkylation repair homolog protein 1 [ALKBH1], YTH domain family 2 [YTHDF2], YTHDF3, and heterogeneous nuclear ribonucleoprotein [HNRNPC]) m^6^A‐related genes were found in islet samples of T2DM patients. A U‐shaped association existed between serum IGF2BP3 levels and odds of T2DM according to cubic natural spline analysis models, after adjustment for body mass index, waist circumference, diastolic blood pressure, total cholesterol, and triglyeride. Multivariate logistic regression showed that progressively higher odds of T2DM were observed when serum IGF2BP3 levels were below 0.62 ng/mL (odds ratio 3.03 [95% confidence interval 1.23–7.47]) in model 4.

**Conclusion:**

Seven significantly altered m^6^A RNA methylation genes were identified in T2DM. There was a U‐shaped association between serum IGF2BP3 levels and odds of T2DM in the general Chinese adult population. This study provides important evidence for further examination of the role of m^6^A RNA methylation, especially serum IGF2BP3 in T2DM risk assessment.

## Graphical abstract

**Highlights**

- This is the first cross‐sectional study of the association between serum IGF2BP3 levels and odds of T2DM in high‐risk Chinese population.
- Two upregulated (IGF2BP2 and IGF2BP3) and 5 downregulated (METTL3, ALKBH1, YTHDF2, YTHDF3, and HNRNPC) m^6^A‐related genes could contribute to the occurrence of T2DM.
- A U‐shaped association existed between serum IGF2BP3 levels and odds of T2DM, which highlights diagnostic and prognostic values of serum IGF2BP3 in T2DM risk assessment.

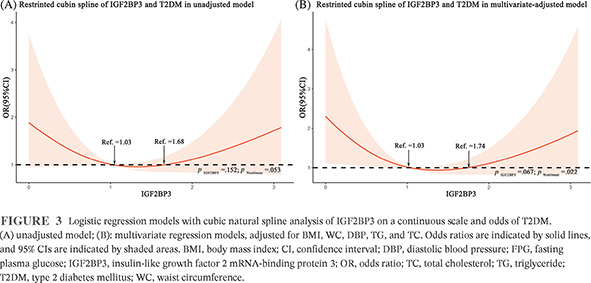

## 摘要

**目的:**探讨N6 ‐甲基腺苷(m^6^A)调节因子在2型糖尿病(T2DM)发病机制中的表达。我们进一步探索血清胰岛素样生长因子2 mRNA结合蛋白3 (IGF2BP3)水平与高危人群T2DM发病风险的关系。

**方法:**从基因表达综合数据库获得基因表达数据集GSE25724, 使用R包ComplexHeatmap生成聚类热图。采用非配对T检验分析13个m^6^A RNA甲基化调控因子在非糖尿病对照者和T2DM受试者之间的差异表达。本研究为横断面研究, 共纳入393例研究对象, 其中新诊断T2DM患者131例, 年龄、性别相匹配的糖尿病前期患者131例, 健康对照者131例。采用限制性立方样条和logistic回归模型分析血清IGF2BP3浓度与T2DM的关系。

**结果:**在T2DM患者胰岛中发现2个上调的m^6^A相关基因(IGF2BP2和IGF2BP3)和5个下调的m^6^A相关基因(METTL3、ALKBH1、YTHDF2、YTHDF3和HNRNPC)。校正BMI、腰围、舒张压、总胆固醇和三酰甘油后, 三次自然样条模型显示血清IGF2BP3水平与T2DM患病风险呈U型曲线关系。多因素logistic回归模型4显示, 当血清IGF2BP3水平低于0.62 ng/ml时, T2DM的发病风险逐渐增加[OR 3.03 (95% CI 1.23 ~ 7.47)]。

**结论:**T2DM患者存在7个m^6^A RNA甲基化基因的显著改变。中国成年人血清IGF2BP3水平与T2DM发病风险之间存在U型关联。本研究为进一步探讨m^6^A RNA甲基化尤其是血清IGF2BP3在T2DM风险评估中的作用提供了重要依据。

## 1 BACKGROUND

Because of the increasing prevalence and diabetes‐related health expenditure, diabetes mellitus and its complications have become a major health threat worldwide.^1^ Approximately 463 million people worldwide had diabetes mellitus in 2019, and this number is expected to grow to approximately 578 million by 2030.^2^ In China, approximately 11 in 100 adults have diabetes mellitus, and 90% of those have type 2 diabetes mellitus (T2DM).^3^ Because T2DM is a complex metabolic disorder caused by the joint effect of genetic and environmental factors, a molecular biomarker that can identify high‐risk individuals for T2DM must be explored.

Epigenetic abnormalities are considered to be linked to obesity, diabetes, and other metabolic diseases. Although it has been widely established that DNA and proteins are regulated by epigenetic mechanisms, RNA modification remains unclear. N6‐methyladenosine (m^6^A) modification as the predominant internal transcriptome modification in eukaryotic messenger RNA (mRNAs), is a dynamic and reversible modification that depends on m^6^A methylation, demethylation, and recognition enzymes – writers (methyltransferase‐like 14 [METTL14], METTL3, and Wilms' tumor 1‐associated protein [WTAP]), erasers (alkylation repair homolog protein 1 [ALKBH1], ALKBH5, and fat‐mass and obesity‐associated protein [FTO]), readers (YTH domain family 1–3 [YTHDF1–3], heterogeneous nuclear ribonucleoproteins [HNRNPs], and insulin‐like growth factor 2 mRNA‐binding proteins 1–3 [IGF2BP1–3]).^4^, ^5^ In particular, numerous experiments have shown that m^6^A plays a key role in many pathological processes in T2DM.^6^, ^7^, ^8^ Studies have demonstrated that METTL14 and METTL3 are downregulated in islet cells of T2DM patients compared to healthy individuals. β‐cell–specific METTL14 knockout mice exhibited reduced β‐cell proliferation, insulin degranulation, and early‐onset diabetes, similar to those with human T2DM.^9^ A high risk of T2DM was associated with a reduction in m^6^A levels in peripheral blood and an increase in the expression of the demethylase FTO mRNA.^6^, ^10^ Another study reported that hyperglycemia contributed to decreased m^6^A modification of keratinocytes in diabetes.^11^ Despite this, the association between m^6^A RNA methylation regulator profiles and T2DM is not fully understood. In this study, we first investigated and validated human pancreas samples with or without T2DM for mRNA expression profiles of key m^6^A‐related genes from Gene Expression Omnibus data sets.

Furthermore, limited but interesting findings have confirmed that unlike other m^6^A‐related genes, IGF2BP3 can be detected in the serum of cancer patients, which reveals that serum IGF2BP3 probably can be used as a diagnostic and prognostic marker in prostate and renal cancers.^12^, ^13^

When assessing disease risk, blood samples have unmatched advantages over tissue‐based analyses. However, to the best of our knowledge, circulating blood levels of IGF2BP3 have not yet been examined in T2DM. Therefore, we further analyzed the serum concentrations of IGF2BP3 in serum samples using enzyme‐linked immunosorbent assay (ELISA), aiming to elucidate the potential role of serum IGF2BP3 level on risk assessment of new‐onset T2DM.

## 2 METHODS

### 2.1 Data set acquisition and selection of m^6^A RNA methylation regulators

The gene expression data set GSE25724 was obtained from the Gene Expression Omnibus data sets (https://www.ncbi.nlm.nih.gov/geo/). The platform for GSE25724 is GPL96 [HG‐U133A] Affymetrix Human Genome U133A Array, which consists of 13 samples—seven islets from nondiabetic pancreases and six islets from diabetic pancreases. Human islets were obtained from organ donors, digested with collagenase, purified by density, and then hand‐picked and cultivated for 2 days in M199 medium. Series Matrix File(s) of download family, GSE25724_RAW.tar of Supplementary file and annotation file of GPL96 were downloaded. Thirteen widely recognized m^6^A RNA methylation regulators from the gene list were selected after converting the gene ID into an official gene symbol. Expression data were obtained by analyzing these CEL files. After normalizing the data, differential expression analysis for 13 m^6^A RNA methylation regulators between nondiabetic controls and T2DM subjects was performed using an unpaired *t* test. A cluster heatmap was generated by using the R package ComplexHeatmap.

### 2.2 Study populations

**FIGURE 1:**
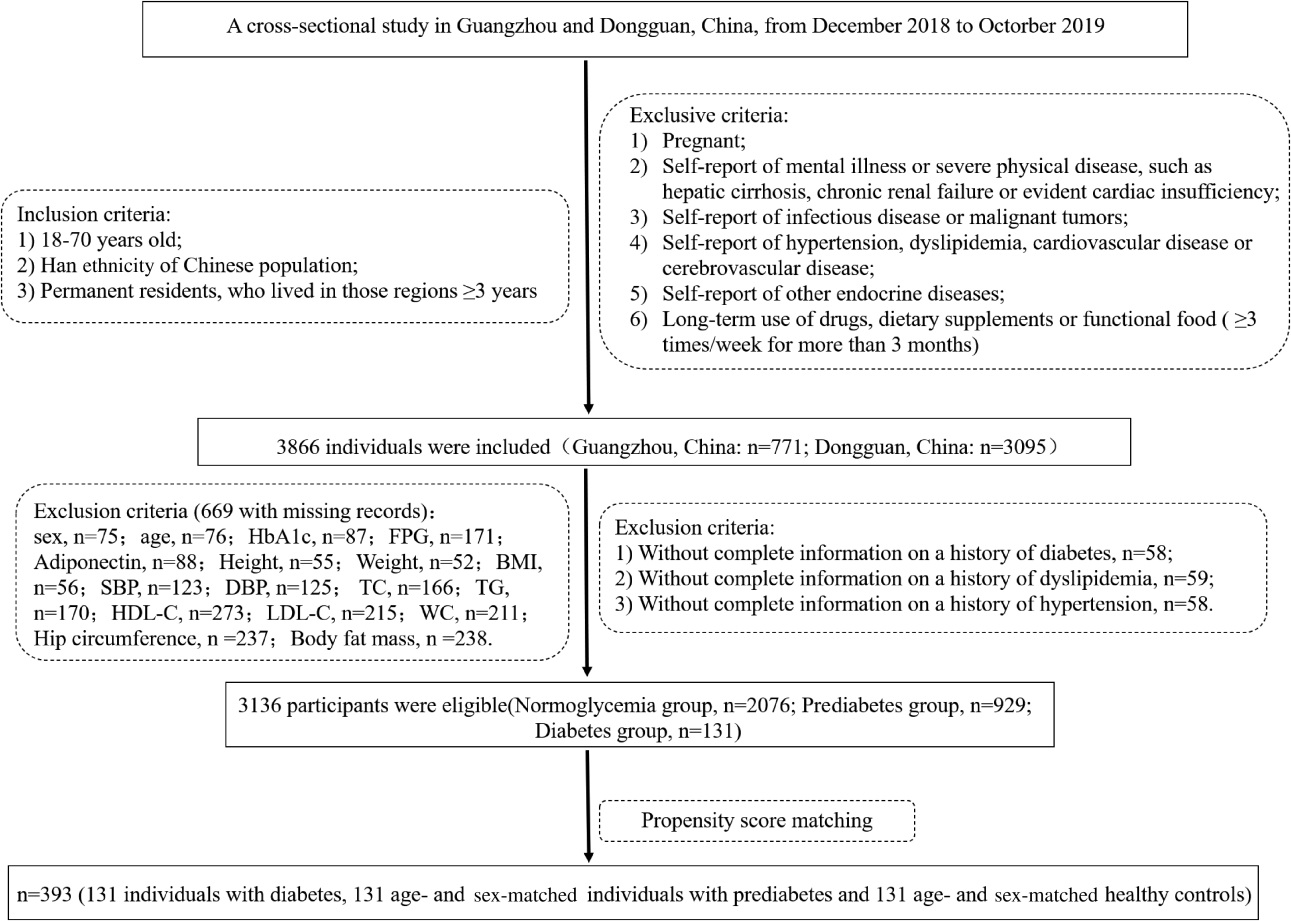
Study population flow chart. BMI, body mass index; DBP, diastolic blood pressure; FPG, fasting plasma glucose; HbA1c, glycosylated hemoglobin; HDL‐C, high‐density lipoprotein cholesterol; LDL‐C, low‐density lipoprotein cholesterol; SBP, systolic blood pressure; TC, total cholesterol; TG, triglyceride; WC, waist circumference.

A cross‐sectional study was conducted in community populations of Guangzhou and Dongguan from December 2018 to October 2019. Our previous paper described the study design and recruitment of participants in detail.^14^ The inclusion criteria, exclusion criteria, and flow diagram of the screening process are shown in Figure 1. In total, 3866 individuals were recruited, in which 669 of those with missing records (sex, *n* = 75；age, *n* = 76；glycosylated hemoglobin [HbA1c], *n* = 87；fasting plasma glucose [FPG], *n* = 171；adiponectin, *n* = 88；height, *n* = 55；weight, *n* = 52；body mass index [BMI], *n* = 56；systolic blood pressure [SBP], *n* = 123；diastolic blood pressure [DBP], *n* = 125；total cholesterol [TC], *n* = 166；triglyeride [TG], *n* = 170；high‐density lipoprotein cholesterol [HDL‐C], *n* = 273；low‐density lipoprotein cholesterol [LDL‐C], *n* = 215；waist circumference [WC], *n* = 211；hip circumference [HC], *n* = 237；body fat mass, *n* = 238) and 61 of those with missing information on a history of diabetes, dyslipidemia, or hypertension were excluded. The final analysis included 3136 individuals, including 131 with new‐onset T2DM (FPG 7.0 mmol/L or HbA1c 6.5%). To decrease the variation among groups and increase the comparability among groups, sex and age were used to derive a propensity score with the matchit() function of the MatchIt package in RStudio. We calculated the odds of T2DM using a logit model that included sex and age as potential indices, and the propensity score ranged from 0 to 1. A propensity score matching analysis was performed after further screening to match each T2DM patient to a prediabetic and a normal blood glucose individual in this cohort based on propensity score. As a final step, 131 prediabetic and 131 normoglycemic subjects were selected from the prediabetes and normoglycemia groups based on their age and gender. Serum IGF2BP3 measurement was performed in a total of 393 subjects. Missing data and extreme values of IGF2BP3 were deleted, and 356 individuals were ultimately included in the following analysis. The current study was approved by the Ethics Committee of Sun Yat‐sen Memorial Hospital, Sun Yat‐sen University. All informed consent forms were signed before data collection.

### 2.3 Measurements

Anthropometric assessments were conducted by well‐trained staff and a standard questionnaire was filled out, which contained information about date of birth, gender, past medical history, present illness, and history of drug or dietary supplementation. Measurements of body height and weight took place with the subjects wearing light clothing and taking off their shoes. BMI was calculated as weight (kg)/height squared (m^2^). WC and HC were measured with subjects standing and breathing steadily. The waist‐to‐hip ratio (WHR) was defined as the ratio of WC to HC. After 5 min of rest, an automated electronic device measured systolic and diastolic blood pressure (OMRON Model HEM‐752 FUZZY; Omron Company).

During laboratory tests, whole blood samples were collected from the vena cava and stored at −80°C following an overnight fast of at least 10 h. The measurement of TC, TGs, LDL‐C, HDL‐C, and FPG were performed with an autoanalyzer (Beckman CX‐7 Biochemical Autoanalyzer). High‐performance liquid chromatography (Bio–Rad) was used to detect the level of HbA1c. Adiponectin was assessed by latex‐enhanced immunoturbidimetry (Uniten Biotechnology Company, Catalog number: 20182400947) according to the manufacturer's instructions. Serum IGF2BP3 was measured using an enzyme‐linked immunosorbent assay (Novus Biologicals, NBP2‐82216) according to the manufacturer's instructions. This assay was sensitive at 0.19 ng/mL and measurable between 0.31 and 20 ng/mL with inter‐ and intra‐assay coefficients of variation of 5% and 6%, respectively.

### 2.4 Definition of diabetes, prediabetes, hypertension, central obesity, and hyperlipidemia

Diagnosis for diabetes (impaired glucose tolerance, defined as 2‐h oral glucose tolerance levels >11.1 mmoL/L, impaired fasting glucose, defined as fasting glucose levels >7.0 mmoL/L and HbA1c >6.5%) and prediabetes mellitus (impaired glucose tolerance, defined as 2‐h oral glucose tolerance levels 7.8–11.0 mmoL/L, impaired fasting glucose, defined as fasting glucose levels 5.6–6.9 mmoL/L and HbA1c 5.7–6.4%) were based on 2020 American Diabetes Association Standards.^15^ General obesity was defined by BMI ≥28.0 kg/m^2^; Central obesity was defined by waistline ≥80 cm for women and waistline ≥85 cm for men. Several criteria were used to define hyperlipidemia: TC ≥5.18 mmol/L, TG ≥1.70 mmol/L, HDL‐C <1.04 mmol/L, or LDL‐C ≥3.37 mmol/L.

### 2.5 Statistical analysis

Continuous variables with a normal distribution were expressed as mean ± SD. We used a one‐way analysis of variance to analyze differences among groups, and Bonferroni corrections were used to determine post hoc comparisons. Data on skewed continuous variables were presented as the median and interquartile range, and Kruskal–Wallis test was applied to compare group differences. To examine the differences between groups, the *X* ^2^ test was applied and categorical variables were specified as numbers (proportions). The serum levels of IGF2BP3 were categorized by quartiles (Q1–4). Unadjusted and multivariate logistic regression analyses were conducted to determine the risk factors for elevated T2DM, along with odds ratios (ORs) and 95% confidence intervals (CIs) for each. Prediabetes subjects were excluded in logistic regression analysis models. Model 1 was unadjusted. Model 2 was adjusted for BMI and WC. Model 3 was further adjusted for DBP. Model 4 was further adjusted for TG and TC. Restricted cubic splines visualized the unadjusted and multivariate dose–response association between IGF2BP3 and diabetes odds. After stratification by restricted cubic spline regression analysis, logistic regression models were performed to assess the associations between T2DM and serum IGF2BP3 concentrations in various exploratory subgroups while adjusting for BMI, WC, DBP, TG, and TC. The confounding factors included in the multivariate‐adjusted logistic regression analysis and restricted cubic spline regression analysis were selected based on previous publications and potential risk factors that associated with the occurrence of T2DM.^14^, ^16^, ^17^ A two‐tailed *p* < .05 was considered as statistically significant. All of statistical analyses in our study were calculated by RStudio version 3.6.1.

## 3 RESULTS

### 3.1 Expression of m^6^A RNA methylation regulators in T2DM

**FIGURE 2:**
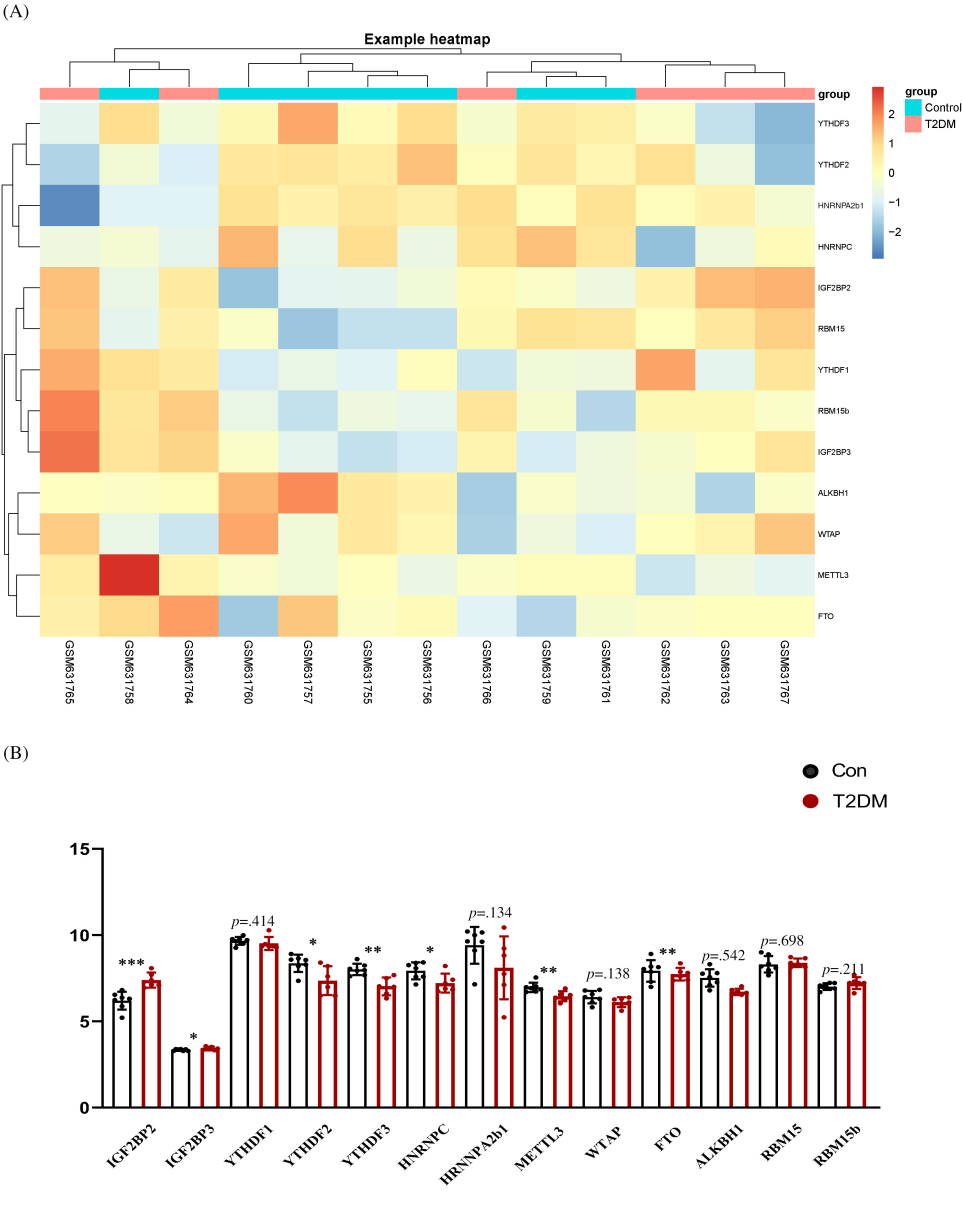
13 m^6^A RNA methylation regulators in islets of T2DM patients and healthy controls. (A) Heatmap; (B) Comparison of mRNA expression of 13 m^6^A RNA methylation regulators. The data are presented as the mean ± SD. *p < 0.05 and **p < 0.01. ALKBH1, alkylation repair homolog protein 1; FTO, fat‐mass and obesity‐associated protein; HNRNPC, heterogeneous nuclear ribonucleoprotein C; HNRNPA2b1, heterogeneous nuclear ribonucleoprotein A2/B1; IGF2BP, insulin‐like growth factor 2 mRNA‐binding protein; METTL3, methyltransferase‐like 3; RBM15, RNA binding motif protein 15; T2DM, type 2 diabetes mellitus; WTAP, Wilms' tumor 1‐associated protein; YTHDF, YTH domain family.

Clustering was performed with Euclidean distance, and the gene expression of 13 m^6^A RNA methylation regulators between T2DM samples and control samples is shown by the heatmap in Figure 2A. As shown in Figure 2B, 2 upregulated (IGF2BP2 and IGF2BP3) and 5 downregulated (METTL3, ALKBH1, YTHDF2, YTHDF3, and heterogeneous nuclear ribonucleoprotein C [HNRNPC]) m^6^A‐related genes in T2DM samples were found to be significantly associated with the occurrence of T2DM compared to nondiabetes samples.

### 3.2 Baseline characteristics of study participants

By referring to related data, we found that most m^6^A RNA methylation regulators are mainly expressed in the cell nucleus and cannot be secreted into serum.^18^ However, IGF2BP3 is free of the aforementioned genetic defects. Thus, IGF2BP3 was the only gene of interest we chose for further study. We aimed to explore IGF2BP3 serum levels and clinical characteristics in 113 T2DM patients, 124 age‐ and sex‐matched participants with prediabetes and 119 age‐ and sex‐matched healthy participants as controls. Table 1 shows the clinical and biochemical characteristics of the 356 individuals included in the final analysis. Participants with T2DM were more likely to have higher weight, BMI, WC, HC, WHR, and TG and to have lower levels of adiponectin than individuals in the prediabetes group and healthy control group.

**TABLE 1:** Clinical and biochemical characteristics of study subjects.

Characteristics	Group			
	Normal	Pre‐DM	DM	*p*_difference_
*N* (%)	119 (33.43)	124 (34.83)	113 (31.74)	—
Male, *n* (%)	63 (52.9%)	64 (51.6%)	60 (53.1%)	.968
Age, years [mean (SD)]	54.3 (10.9)	53.8 (11.4)	53.7 (11.7)	.898
Height, cm [mean (SD)]	160 (8.46)	160 (8.93)	161 (9.93)	.699
Weight, kg [mean (SD)]	59.7 (11.2)	63.8 (11.3)^#^	67.3 (13.4)^#^	<.001
BMI, kg/m^2^ [mean (SD)]	23.2 (3.13)	24.7 (3.53)^#^	25.9 (3.96)^#^ ^,^ *	<.001
WC, cm [mean (SD)]	81.5 (9.28)	85.0 (8.26)^#^	88.2 (10.5)^#^ ^,^ *	<.001
HC, cm [mean (SD)]	93.8 (6.81)	95.8 (5.94)	97.7 (7.60)^#^	<.001
WHR, [mean (SD)]	0.87 (0.06)	0.89 (0.06)	0.90 (0.07)^#^	<.001
SBP, mmHg [mean (SD)]	121 (12.6)	124 (12.8)	126 (18.6)	.066
DBP, mmHg [mean (SD)]	74.0 (8.66)	75.4 (8.26)	77.8 (11.5)^#^	.010
TC, mmol/L [mean (SD)]	5.28 (0.93)	5.49 (1.00)	5.62 (1.16)^#^	.040
TG, mmol/L [median (Q1; Q3)]	1.17 [0.90;1.64]	1.36 [0.94;1.92]^#^	1.78 [1.27;2.60]^#^	<.001
HDL‐C, mmol/L [mean(SD)]	1.47 (0.42)	1.48 (0.45)	1.48 (0.71)	.978
LDL‐C, mmol/L [mean(SD)]	3.19 (0.74)	3.30 (0.81)	3.33 (1.06)	.430
Adiponectin, mg/L [median (Q1; Q3)]	4.50 [3.00;6.45]	5.10 [3.10;6.43]^#^	3.20 [2.10;4.80]^#^	<.001
IGF2BP3, [median (Q1; Q3)]	1.07 [0.76;1.65]	1.07 [0.63;1.83]	0.97 [0.53;1.79]	.794

Abbreviations: BMI, body mass index; DBP, diastolic blood pressure; DM, diabetes mellitus; HC, hip circumference; HDL‐C, high‐density lipoprotein cholesterol; IGF2BP3, insulin‐like growth factor 2 mRNA‐binding proteins 3; LDL‐C, low‐density lipoprotein cholesterol; SBP, systolic blood pressure; TC, total cholesterol; TG, triglycerides; WC, waist circumference; WHR, waist to hip ratio.

^#^ *p* < .05 versus normal group.

* *p* < .05 comparing between: pre‐DM group and DM group.

In addition, we divided the serum IGF2BP3 into quartiles and displayed the baseline characteristics of the study participants by quartiles of serum IGF2BP3 in Table 2. Compared with the lowest serum IGF2BP3 quartile, serum IGF2BP3 concentrations in the higher quartiles were positively associated with older age (*p* < .001). In addition, compared with quartile 3, participants in other quartiles had higher levels of HbA1c (*p* < .016).

**TABLE 2:** Clinical characteristic of participants according to quartiles of serum IGF2BP3 levels.

	Q1	Q2	Q3	Q4	*p*_difference_
*N* (%)	91 (25.56)	88 (24.44)	89 (25.28)	88 (24.72)	—
Distribution, [min, max]	[0, 0.62]	(0.62, 1.04]	(1.04, 1.76]	(1.76, 3.64]	—
DM	37(41%)	23(26%)	23(26%)	30(34%)	—
Pre‐DM	31(34%)	29(33%)	32(36%)	32(36%)	—
Normal glucose tolerance	23(25%)	36(41%)	34(38%)	26(30%)	—
IGF2BP3, [Median (Q1; Q3)]	0.35 [0.08;0.48]	0.85 [0.72;0.92]	1.31 [1.15;1.48]	2.26 [1.98;2.56]	<.001
Male, *n* (%)	54(59.3%)	51(58.6%)	46(51.1%)	36(40.9%)	.050
Age, years [mean (SD)]	57.7(10.7)	55.0(11.2)	53.0(11.3)	49.9(10.7)	<.001
Height, cm [mean (SD)]	160(8.84)	161(8.74)	161(8.85)	160(10.0)	.932
Weight, kg [mean (SD)]	63.8(13.0)	62.8(12.8)	63.6(12.7)	64.0(11.0)	.931
BMI, kg/m^2^ [mean (SD)]	24.7(4.11)	24.2(3.35)	24.4(3.76)	25.0(3.53)	.495
WC, cm [mean (SD)]	85.7(10.1)	84.2(9.86)	84.6(10.1)	85.0(8.75)	.768
HC, cm [mean (SD)]	96.0(7.49)	95.2(6.32)	95.5(7.55)	96.2(6.42)	.809
WHR, [mean (SD)]	0.89(0.06)	0.88(0.07)	0.88(0.07)	0.88(0.06)	.768
SBP, mmHg [mean (SD)]	125(15.9)	123(14.3)	124(15.0)	122(14.3)	.568
DBP, mmHg [mean (SD)]	76.8(10.6)	75.6(8.72)	74.9(9.01)	75.5(10.1)	.622
TC, mmol/L [mean (SD)]	5.41(1.02)	5.42(0.94)	5.42(1.11)	5.60(1.08)	.555
TG, mmol/L [median (Q1; Q3)]	1.29[0.90;1.90]	1.43 [0.94;2.10]	1.49 [1.09;2.27]	1.40 [1.10;2.24]	.164
HDL‐C, mmol/L [mean (SD)]	1.51(0.68)	1.49(0.47)	1.41(0.33)	1.50(0.60)	.520
LDL‐C, mmol/L [mean (SD)]	3.17(0.95)	3.31(0.82)	3.26(0.82)	3.36(0.89)	.523
FPG, mmol/L [mean (SD)]	5.95(2.15)	5.69(2.09)	5.86(2.05)	5.87(1.73)	.857
HbA1c, % [mean (SD)]	6.45(1.55)	6.05(1.16)	5.86(0.78)	6.04(1.42)	.016
Adiponectin, mg/L [median (Q1; Q3)]	4.20 [2.95;6.40]	4.50 [3.00;6.30]	4.70 [2.75;6.07]	3.65 [2.50;5.15]	.068

Abbreviations: BMI, body mass index; DBP, diastolic blood pressure; DM, diabetes mellitus; FPG, fasting plasma glucose; HbA1c, glycosylated hemoglobin; HC, hip circumference; HDL‐C, high‐density lipoprotein cholesterol; IGF2BP3, insulin‐like growth factor 2 mRNA‐binding proteins 3; LDL‐C, low‐density lipoprotein cholesterol; SBP, systolic blood pressure; TC, total cholesterol; TG, triglycerides; WC, waist circumference; WHR, waist to hip ratio.

### 3.3 U‐shaped associations between odds of T2DM and serum IGF2BP3 quartiles

**FIGURE 3:**
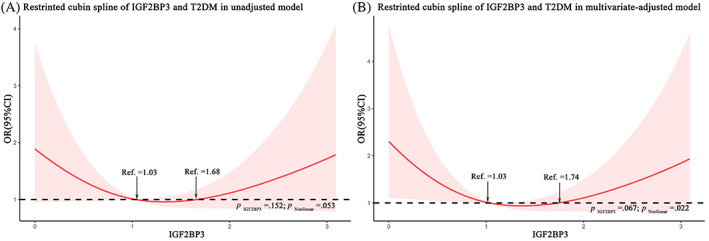
Logistic regression models with cubic natural spline analysis of IGF2BP3 on a continuous scale and odds of T2DM. (A) unadjusted model; (B): multivariate regression models, adjusted for BMI, WC, DBP, TG, and TC. Odds ratios are indicated by solid lines, and 95% CIs are indicated by shaded areas. BMI, body mass index; CI, confidence interval; DBP, diastolic blood pressure; FPG, fasting plasma glucose; IGF2BP3, insulin‐like growth factor 2 mRNA‐binding protein 3; OR, odds ratio; TC, total cholesterol; TG, triglyceride; T2DM, type 2 diabetes mellitus; WC, waist circumference.

To visually demonstrate our analysis, a restricted cubic spline regression line was performed and presented the association between serum IGF2BP3 levels and odds of T2DM as a U shape on a continuous scale (*p* for nonlinearity = .053), as shown in Figure 3A. After adjusting for BMI, WC, DBP, TC, and TG, the *p* value for the test of nonlinearity hypotheses was .022, confirming the significant nonlinear relationship between IGF2BP3 levels and odds of T2DM (Figure 3B). Consistently, according to cubic natural spline analysis models, the odds of T2DM were lower with increasing IGF2BP3 levels in participants with serum IGF2BP3 levels <1.03 ng/mL (ng/ml: OR 1.99 [95% CI 0.96–4.11]) and higher with increasing IGF2BP3 levels in those with serum IGF2BP3 levels >1.74 ng/mL (ng/ml: OR 1.97 [95% CI 0.90–4.28]) in Model 4 (Table 3).

**TABLE 3:** Relations between serum IGF2BP3 levels and odds of T2DM.

Models	IGF2BP3 (stratified by restricted cubic spline regression analysis)			
	<1.03	[1.03;1.74]	>1.74	*p*_trend_
*N* (%)	102 (44.35)	61 (26.52)	67 (29.13)	
Model 1	1.86 (0.97, 3.55)	1	1.81 (0.89, 3.66)	.061
Model 2	1.76 (0.88, 3.54)	1	1.87 (0.88, 3.95)	.110
Model 3	1.71 (0.85, 3.44)	1	1.88 (0.89, 3.99)	.133
Model 4	1.99 (0.96, 4.11)	1	1.97 (0.90, 4.28)	.064

Models	IGF2BP3 (stratified by quartiles [Q1–Q4])				
	Q1	Q2	Q3	Q4	*p*_trend_
*N* (%)	48 (21.82)	59 (26.82)	57 (25.91)	56 (25.45)	—
Distribution	[0,0.62]	(0.62,1.04]	(1.04,1.76]	(1.76,3.64]	—
Model 1	2.96 (1.33, 6.58)	0.94 (0.45, 1.99)	1	1.71 (0.81, 3.59)	.160
Model 2	2.73 (1.15, 6.48)	1.01 (0.46, 2.23)	1	1.86 (0.84, 4.09)	.125
Model 3	2.58 (1.08, 6.18)	0.99 (0.45, 2.20)	1	1.87 (0.85, 4.12)	.122
Model 4	3.03 (1.23, 7.47)	1.09 (0.48, 2.48)	1	1.99 (0.87, 4.51)	.101

*Note*: Model 1: unadjusted; Model 2: adjusted by BMI + WC; Model 3: adjusted by BMI + WC + DBP; Model 4: adjusted by BMI + WC + DBP + TG + TC.

Abbreviations: BMI, body mass index; DBP, diastolic blood pressure; IGF2BP3, insulin‐like growth factor 2 mRNA‐binding proteins 3; TC, total cholesterol; TG, triglycerides; T2DM, type 2 diabetes mellitus; WC, waist circumference.

Because of the U‐shaped association between serum IGF2BP3 levels and odds of T2DM, we further used unadjusted and multivariate logistic regression to assess the association between IGF2BP3 levels and odds of incident T2DM, with quartile 3 of serum IGF2BP3 concentrations as a reference. As shown in Table 3, both high and low levels of IGF2BP3 resulted in increased odds of T2DM. Progressively higher odds of T2DM were observed when the serum IGF2BP3 level was below 1.04 ng/mL (ng/ml: OR 1.09 [95% CI 0.48–2.48]) in quartile 2 and over 1.76 ng/mL (ng/ml: OR 1.99 [95% CI 0.87–4.51]) in quartile 4 after adjustment for BMI, WC, DBP, TC, and TG in Model 4. Notably, individuals in the lowest IGF2BP3 quartile had 3.03 times higher odds of T2DM than individuals in the reference quartile (OR 3.03 [95% CI 1.23–7.47]) in the multivariate‐adjustment Model 4 (Table 3).

### 3.4 Stratified analyses by potential effect modifiers

We further performed exploratory subgroup analyses to assess the association between T2DM and serum IGF2BP3 concentrations, stratified by restricted cubic spline regression analysis. As shown in Table 4, the robustness of the association was confirmed, and similar U‐shaped relationships were found for serum IGF2BP3 concentrations with T2DM in various subgroups.

**TABLE 4:** Relations between serum IGF2BP3 levels and odds of T2DM in subgroups suggested by restricted cubic spline regression analysis.

Subgroup		IGF2BP3			
		<1.03	[1.03;1.74]	>1.74	*p*_trend_
*N* (%)	Cases (%)	102 (44.35)	61 (26.52)	67 (29.13)	
Hypertension					1.000
No	217	1.73 (0.83, 3.60)	1	1.89 (0.86, 4.18)	
Yes	13	1119.14 (0.00, Inf)	1	0.00 (0.00, Inf)	
Central‐obesity					.260
No	96	1.03 (0.31, 3.36)	1	1.81 (0.55, 5.92)	
Yes	134	3.26 (1.22, 8.74)	1	2.21 (0.79, 6.20)	
Obesity					.833
No	192	2.21 (0.99, 4.94)	1	2.09 (0.92, 4.77)	
Yes	38	1.56 (0.14, 17.70)	1	0.74 (0.03, 18.69)	
Hyperlipidemia					.487
No	180	2.17 (0.93, 5.05)	1	1.70 (0.69, 4.17)	
Yes	50	1.69 (0.36, 7.81)	1	3.11 (0.44, 22.17)	

*Note*: Adjusted by BMI + WC + DBP + TG + TC.

Abbreviations: BMI, body mass index; DBP, diastolic blood pressure; IGF2BP3, insulin‐like growth factor 2 mRNA‐binding proteins 3; TC, total cholesterol; TG, triglycerides; T2DM, type 2 diabetes mellitus; WC, waist circumference.

## 4 DISCUSSION

In the current study, we determined that YTHDF2, YTHDF3, HNRNPC, METTL3, and ALKBH1 mRNA expression was downregulated in T2DM islets. In addition, IGF2BP2 and IGF2BP3 mRNA levels were also shown to be increased in islets of T2DM patients compared to controls. In contrast, a previous study revealed that mRNA expression levels of several m^6^A modulators were decreased in the whole islets of T2DM patients compared to controls, including YTHDF1, YTHDF3, METTL14, FTO, pancreatic and duodenal homeobox 1 (PDX1), and ALKBH5. Another prior study reported that METTL3, METTL14, and WTAP mRNA expression levels of white blood cells were significantly higher in patients with T2DM than in the controls.^6^ These inconsistent findings might be due to high sample variability and small sample size. Together, these data revealed that m^6^A modulators might be involved in the pathogenesis of T2DM. Numerous studies have underscored the significance of m^6^A RNA modification in metabolism‐related human diseases, such as obesity,^19^, ^20^ nonalcoholic fatty liver disease,^21^, ^22^ hypertension,^23^ and osteoporosis.^24^ However, knowledge of m^6^A modification remains preliminary in the diabetes field, especially in T2DM.^6^, ^7^, ^8^, ^9^, ^10^ For instance, a previous study found that the m^6^A methyltransferase METTL14 contributed to an increased risk of gestational DM development via enhancement of transforming growth factor‐β signaling and downregulation of Wnt signaling.^25^ METTL14 deficiency has been also demonstrated to decreased insulin secretion and lead to glucose intolerance.^8^ Xie et al illustrated the critical roles for the m^6^A writer METTL3‐mediated m^6^A modification in improving glucose homeostasis and enhancing insulin sensitivity in T2DM.^7^ In addition to m^6^A writers, various types of m^6^A readers and their protein partners play a determinant role in the dynamic regulation of m^6^A modification. Some of them have been identified as potential biomarkers and therapeutic targets for T2DM. YTHDF2, a reader of m^6^A modification, was reported to be negatively associated with the risk of T2DM. The downregulation of YTHDF2 in the pancreatic islets of a prediabetic mouse model could be restored by the insulin sensitizer pioglitazone.^26^ Thus, further studies remain to be developed to elaborate the prediction value of other m^6^A effector proteins in T2DM, which may provide a better understanding of glucose metabolism and find more potential therapeutic targets.

However, most of the m^6^A modulators predominantly localize to the nucleus (METTL3, METTL14, YTHDC1) or the cytoplasm (YTHDF1‐3) of eukaryotic cells.^18^ Unlike the m^6^A modulators mentioned previously, IGF2BP3 has been proven to be secreted into serum and could be a promising diagnostic and prognostic marker of prostate cancer and renal cell carcinoma.^12^, ^13^ These results remind us that IGF2BP3 may be an effective circulating biomarker for disease risk assessment. Thus, we further detected the serum concentration of IGF2BP3 in a population‐based cross‐sectional study and found that there was a nonlinear, U‐shaped association between serum IGF2BP3 level and odds of T2DM, with a minimal risk at 0.62 to 1.76 ng/L of serum IGF2BP3 level. The correlation remained stable after adjusting for potential confounders and performing subgroup analyses based on hypertension, central obesity, obesity, and hyperlipidemia. To the best of our knowledge, this is the first study to investigate the association between serum IGF2BP3 and type 2 diabetes risk in subjects with a high risk of T2DM, offering some novel interesting clues for the clinical predictive value of serum IGF2BP3 in T2DM.

Studies have shown that IGF2BPs (IGF2BP1–3) are enriched for genes related to energy metabolism, including lipogenesis, glucose tolerance, and oxidative respiration.^27^ For example, IGF2BP1 contributes to adipocyte proliferation and fat metabolism by promoting IGF2 protein expression.^28^ IGF2BP2 was also confirmed to play modulatory roles in adipocyte differentiation and lipid accumulation.^29^ A combination study of genomic and transcriptomic analysis presented a positive correlation between the circulating transcript of IGF2BP2 and fasting insulin in individuals without diabetes, suggesting that IGF2BP2 might take part in the pathophysiology of T2DM before the onset of the disease.^30^ A genome‐wide association study also reported that the estimated risk of T2DM for individuals with the risk allele in rs4402960 (IGF2BP2) was three times higher than those without this risk allele.^31^ Another study found that the modulation of IGF2BP2 expression in patients with high glucose levels can improve glucose tolerance.^32^ Together, these studies revealed the indispensable roles of IGF2BP2 in metabolism, including mediating glucose tolerance, insulin sensitivity, and energy consumption. However, relatively speaking, the function and mechanism of IGF2BP3 in metabolism have not been well explained. Our study provides evidence that there is a nonlinear, U‐shaped association between serum IGF2BP3 levels and the odds of T2DM, suggesting a possible regulatory mechanism that maintains the balance of glucose metabolism and serum IGF2BP3 concentrations in individuals with a high risk of T2DM.

The underlying mechanisms whereby there is a U‐shaped association between serum IGF2BP3 levels and odds of T2DM have not yet been elucidated. IGF2BP3 has been reported to be upregulated in many types of tumors, including pancreatic cancer,^33^ lung adenocarcinoma,^34^ triple‐negative breast cancer,^35^ colorectal cancer,^36^ hepatocellular carcinoma,^37^ and gliomas,^38^ suggesting that IGF2BP3 can serve as a novel cancer biomarker for early diagnosis and prognosis. Mechanically, highly expressed IGF2BP3 served a moderating role to promote the proliferation, migration, and glycolysis of endometrial, nasopharyngeal, and liver carcinomas by enhancing the stability of specific genes, such as E2F3,^39^ KPNA2,^40^ and pyruvate dehydrogenase kinase 4 (PDK4).^41^ In addition, IGF2BP3 regulated target genes in an m^6^A‐dependent manner, contributing to cancer progression, cell cycle, angiogenesis, and drug resistance in different types of malignancies, including laryngeal squamous cell carcinoma,^42^ colon cancer,^43^ colorectal cancer,^44^ and triple‐negative breast cancer.^45^ Notably, a significant correlation between high IGF2BP3 protein levels and unfavorable prognosis was found in ovarian carcinoma^46^ of clear cell subtype. However, there was contradictive result suggesting that IGF2BP3 expression was positively associated with improved survival in ovarian cancer.^47^ Differences in ovarian carcinoma subtypes of these studies may be accountable for these contradictory observations, indicating that IGF2BP3 played diverse biological effects in various disease. Hypomethylation in islets of T2DM has been reported to be associated with β‐cell pathophysiology through downregulation of the insulin/IGF1–AKT–PDX1 pathway.^9^ Furthermore, previous studies suggested that IGF2BP3 might be involved in the positive regulation of IGF1R and IGF2 mRNA at the posttranscriptional level, leading to enhancement of cell viability, proliferation, and migration, which could be behind the association between higher IGF2BP3 levels and cancer.^48^ Together, these studies suggest that IGF2BP3 may contribute to T2DM development via the insulin/IGF1R pathway. Specifically, the function of IGF1R in T2DM development was diverse at different periods of T2DM. During the period of prediabetes, the interference of IGF1R signaling in fat tissue may result in insulin resistance and progression to T2DM.^49^ Defects in insulin and IGF1 signaling pathways were also found to be responsible for decompensation of β‐cell proliferation and mass, which contributes to disturbance of insulin secretion and glucose intolerance in T2DM.^50^ These studies indicated that low IGF2BP3 may increase the odds of T2DM through interaction with IGF1 signaling on insulin resistance and β‐cell failure. Nevertheless, the upregulation of IGF1R was observed followed by hyperglycemia and hyperinsulinemia, leading to deterioration of DM.^49^ Together, the interaction of IGF2BP3‐IGF1R and the complex role of IGF1R signaling in T2DM may help us to explain the U‐shaped association between IGF2BP3 levels and the odds of T2DM. Ennajdaoui et al reported that the capability of IGF2BP3 on mRNA stability was bimodal because it could protect mRNA from or enhance degradation.^51^ This mechanism may explain why both higher and lower IGF2BP3 levels were associated with a high risk for T2DM. It will be interesting to further investigate how IGF2BP3 activities are defined in different periods of T2DM development.

### 4.1 Limitations

Several limitations in the current study should be addressed. First, causality between serum IGF2BP3 levels and T2DM cannot be determined, as our study was a cross‐sectional design. However, this study may provide insights into the potential value of serum IGF2BP3 in predicting T2DM occurrence in high‐risk populations. Second, incomplete data compilation may lead to potential interpretation bias of the results. The collection of socioeconomic, lifestyle, comorbidity, and complication data should be considered to strengthen the findings of the present study. Third, the individuals enrolled in the present study were Chinese, so these results may be difficult to generalize to other ethnicities. Fourth, the size of the data set sample and study population is relatively small and the direct evidence of serum IGF2BP3 contribution to overall obesity and central obesity individuals is relatively insufficient. It is difficult to fully explain mechanistically the differences in subgroups by the data available. Large‐scale prospective cohort studies and laboratory experiments are needed to further elucidate such association and the comprehensive molecular mechanism of m^6^A‐related genes, specifically IGF2BP3, in T2DM. Finally, we measured the level of IGF2BP3 only once, and the dynamic observation of IGF2BP3 changes in blood may better identify its association with the development of T2DM.

### 4.2 Future directions

In the present study, we comprehensively analyzed the expression of m^6^A‐regulated genes in T2DM. The strengths of the present study also included a high‐risk population, the application of nonlinear regression analyses instead of a simple linear test, a novel perspective of serum IGF2BP3 on T2DM prediction, and multivariate adjustment for confounding factors, such as BMI, WC, DBP, TC, and TG. However, future studies are still needed to clarify the detailed roles of these RNA methylation regulators in T2DM, such as whether their abnormal altered lead to the development of T2DM as well as whether serum IGF2BP3 was an independent risk factor for T2DM.

## 5 CONCLUSIONS

In summary, we identified several significantly altered m^6^A RNA methylation regulators in T2DM, including IGF2BP2, IGF2BP3, YTHDF2, YTHDF3, HNRNPC, METTL3, and ALKBH1. Among these, a U‐shaped association between serum IGF2BP3 and odds of T2DM in Chinese adults was found, providing an opportunity to examine the continuous association between circulating IGF2BP3 concentrations and T2DM, with adjustments for a number of potential cofounders and a series of subgroup analyses. If further validated, our findings highlight the importance of paying attention to the serum IGF2BP3 levels for T2DM risk assessment in high‐risk populations.

## AUTHOR CONTRIBUTIONS

Meng Ren, Li Yan and Wei Wang conceived and designed the experiments; Shihong Wu, Juying Tang, Yiqin Qi, and Wanting Feng acquired the clinical data; Xiaoying Wu, Wei Wang, Feng Li, and Xiaoyun Zhang performed the experiments; Lili You, Shujin Fan, and Xiaoying Wu analyzed the data; Xiaoying Wu and Wei Wang wrote the manuscript. All authors read and approved the final manuscript.

## CONFLICT OF INTERESTS STATEMENT

The authors have nothing to disclose.

## Data Availability

The data sets generated and/or analyzed during the current study are available from the corresponding author upon reasonable request.
